# Promising Biomolecules with High Antioxidant Capacity Derived from Cryptophyte Algae Grown under Different Light Conditions

**DOI:** 10.3390/biology11081112

**Published:** 2022-07-26

**Authors:** Maryam Abidizadegan, Jaanika Blomster, David Fewer, Elina Peltomaa

**Affiliations:** 1 Microbiology and Biotechnology Programme, Environmental Laboratory, Faculty of Biological and Environmental Sciences, University of Helsinki, 15140 Lahti, Finland; 2 Ecosystem and Environment Research Programme, Faculty of Biological and Environmental Sciences, University of Helsinki, 00014 Helsinki, Finland; jaanika.blomster@helsinki.fi; 3 Department of Microbiology, Faculty of Agriculture and Forestry, University of Helsinki, 00014 Helsinki, Finland; david.fewer@helsinki.fi; 4 Institute of Atmospheric and Earth System Research (INAR)/Forest Sciences, University of Helsinki, 00014 Helsinki, Finland; elina.peltomaa@helsinki.fi

**Keywords:** antioxidant activity, cryptophytes, exopolysaccharides, LED lights, phenolic compounds, phycoerythrin

## Abstract

**Simple Summary:**

In recent decades, the demand for natural and sustainable bioproducts has risen markedly. Accordingly, microalgae have received much attention as a promising biological resource with great industrial potential, since the microalgal production of biologically active compounds can be boosted by changing their cultivation conditions. Light is one of the key factors in the photosynthetic process, which directly affects cell division and the production of biochemical compounds. This study investigated the effect of light color and the species-specific capability of cryptophyte algae to produce phycoerythrin, phenolic compounds, and exopolysaccharides. The produced biomolecules were further studied for their antioxidant activity. The results showed that changes in light quality significantly affect the biochemical compositions of cryptophyte algae. Moreover, species-specific responses to changes in light quality were identified. The quantity and quality of derived biomolecules from the studied cryptophytes are remarkable and indicate that cryptophytes could be considered promising candidates for producing natural biochemical products for practical applications in various industry sectors, such as food, pharmacy, and cosmetics.

**Abstract:**

The accumulation and production of biochemical compounds in microalgae are influenced by available light quality and algal species-specific features. In this study, four freshwater cryptophyte strains (*Cryptomonas ozolinii*, *C. pyrenoidifera*, *C. curvata*, and *C.* sp. (CPCC 336)) and one marine strain (*Rhodomonas salina*) were cultivated under white (control), blue, and green (experimental conditions) lights. Species-specific responses to light quality were detected, i.e., the color of light significantly affected cryptophyte biomass productivity and biochemical compositions, but the optimal light for the highest chemical composition with high antioxidant capacity was different for each algal strain. Overall, the highest phycoerythrin (PE) content (345 mg g^−1^ dry weight; DW) was reached by *C. pyrenoidifera* under green light. The highest phenolic (PC) contents (74, 69, and 66 mg g^−1^ DW) were detected in *C. curvata* under control conditions, in *C. pyrenoidifera* under green light, and in *C. ozolinii* under blue light, respectively. The highest exopolysaccharide (EPS) content (452 mg g^−1^ DW) was found in *C. curvata* under the control light. In terms of antioxidant activity, the biochemical compounds from the studied cryptophytes were highly active, with IC_50_ -values < 50 µg mL^−1^. Thus, in comparison to well-known commercial microalgal species, cryptophytes could be considered a possible candidate for producing beneficial biochemical compounds.

## 1. Introduction

Over the last decade, demands for natural and sustainable bioproducts have greatly increased the need to provide alternative biomass sources for commercial and industrial activities [1,2]. In particular, microalgae have received attention as a promising biological resource for a wide range of chemical compounds with great industrial possibilities [3]. Pigments, polyphenols, and exopolysaccharides are examples of algal chemical compounds with potential applications in the pharmaceutical, nutraceutical, cosmetic, biotechnological, and food sectors. 

Phycoerythrin (PE)—the red-colored photosynthetic pigment from the family of phycobiliproteins—is applied as a food colorant and additive in the food industry, natural dye in cosmeceuticals, and fluorescent probes in biomedical science [4,5]. Another important group of compounds that underscore the importance of microalgal function in industries is phenolic compounds (PC). The antimicrobial activity of PC makes them beneficial as bio-preservatives in the food industry, as it allows them to constrain the oxidation and growth of microorganisms, and hence extend the shelf life of products [6]. Exopolysaccharides (EPS) represent a group of substantial high-molecular-weight biopolymers extracted from microorganisms such as microalgae [7]. The main components of EPS include lipids, proteins, polysaccharides, and nucleic acids [8]. EPS are widely employed as thickeners and gelling additives in food industries to improve the quality of food texture [9]. Importantly, all of the above-specified substances are recognized as antioxidants due to their ability to bind and neutralize free radicals [10,11,12]. Antioxidant properties play a key role in the action against reactive oxygen species (ROS), which lead to many human diseases including type 2 diabetes, cancer, cardiovascular, and chronic kidney diseases [13]. 

Cryptophytes are one of the main groups of phytoplankton, and notable primary producers, in both freshwater and marine ecosystems. They are an extremely rich food source for consumers due to their great fatty acid, sterol, and amino acid profiles that fulfil the needs of consumers [14,15,16,17]. Since cryptophyte algae do not have a heavy cell wall structure formed of silica or cellulose, cell rupturing and processing for commercial purposes are easy [16,18]. Nevertheless, this promising group of algae is practically unexploited in technology. Therefore, evaluating and optimizing their culture conditions is needed to profitably produce biomass and natural bioactive compounds. 

The major light-harvesting pigments of cryptophytes are called phycobiliproteins (PBPs) [19]. In contrast to red algae and cyanobacteria, each cryptophyte strain produces only one type of PBPs: Phycoerythrin or phycocyanin [20]. This removes the need to separate different PBPs from each other and consequently simplifies the protein purification in the PBP production process [21]. All of the species studied in our research contain only PE, and due to its lower molecular weight compared to cyanobacterial and red algal PEs, cryptophyte PE is more practical for use in fluorescent labelling and applications in the food industry [21,22]. The main commercial phycoerythrin producers are the rhodophyta *Porphyridium* and the cyanobacteria *Arthrospira* and *Nostoc* with ~10, 30, and 20% of the maximum dry weight (DW), respectively, grown in industrial wastewater and at low light density [23]. The PE content of the cryptophyte *Rhodomonas salina* has been reported to be 11.5% of DW [24].

Phenolic compounds (PC) are useful in various industrial applications due to their antioxidant activity [25,26,27]. The highest reported PC content of microalgae has been in the cyanobacteria *Nostoc* and *Arthrospira*, as well as in the chlorophyte *Scenedesmus*, with ~6, 4.3, and 4.3% PC of DW, respectively [28]. Although the existence of the flavonoid 2-styrylchromone has been reported in the marine cryptophyte *Chrysophaeum taylori* [29], studies on the PC of cryptophytes are rare. Similarly, exopolysaccharides (EPS) are a group of potential polymers also produced by cryptophytes [30,31]. The most commonly used producers—*Cyanobacterium aponinum*, *Spirulina* sp., and *Nostoc* sp.—have been reported to produce EPS with 6–8.5% [32], 15–45% [33], and 15.5–21% [34] of DW, respectively. However, reports of EPS production by cryptophytes are limited. Only one study showed EPSs derived from *Cryptomonas obovata*, a tropical cryptophyte, including fucose and N-acetyl galactosamine [35]. Therefore, further research on PBPs, PC, and EPS produced by different cryptophyte strains is needed, particularly under different culture conditions. 

The production of bioactive compounds by microalgae is moderated by changing cultivation strategies. Adjustable growth conditions, including light, nutrients, and aeration, form the optimal conditions to enhance biomass, as well as the yield of the compounds and their qualities [1,36]. Light is regarded as a contributing factor in the photosynthetic process, and directly affects the cell division, growth, and production of metabolites [37,38]. Studies have reported the dependency of photosynthesis on light wavelength [39]. Blue wavelengths (430–500 nm) affect the pigmentation, synthesis of secondary metabolites, photosynthetic activity, and development of chloroplast [40]. The orange or red wavelengths (640–670 nm) are effective for improving photosynthetic efficiency via the influence on the development of the photosynthetic system, net photosynthetic rate, and primary metabolism [41], and green wavelengths (500–600 nm) have significant effects on growth and photosynthetic activity [42].

In response to changes in light, microalgae produce ROS, which can be toxic and lead to cell damage. On the other hand, microalgae synthesize compounds with antioxidant properties to scavenge produced ROS [17,18]. As the effect of light on molecule production is complex and species-specific [1], it is necessary to evaluate and optimize the effect of changes in light on the production of new species.

The primary aim of this study was to screen the dry biomass, PE, PC, and EPS production of different cryptophyte strains under manipulated light conditions. Additionally, we measured the effect of light quality on the antioxidant activity of the obtained PE, PC, and EPS. Microalgae are most commonly grown under full-spectrum light (white light) with high intensity. In this study, we assessed the response of cryptophytes to monochrome LED light with low intensity. Accordingly, growth under white light was considered the control condition, while blue and green lights were regarded as experimental treatments. The results obtained in this study are compared to the known species and growth conditions used for the production of bioactive compounds.

## 2. Materials and Methods

### 2.1. Experiment Setup

Four freshwater cryptophyte strains (*Cryptomonas ozolinii* (UTEX LB 2782), *Cryptomonas pyrenoidifera* (CCAP 979/61), *Cryptomonas curvata* (CCAP 979/63), and *Cryptomonas* sp. (CPCC 336) and one marine cryptophyte strain (*Rhodomonas salina* (CCMP 757)) were cultured in 2 L glass bottles for 10 days and harvested during the exponential growth phase. A modified WC medium (MWC: CaCl_2_·2H_2_O, MgSO_4_·7H_2_O, NaHCO_3_, K_2_HPO_4_·3H_2_O, NaNO_3_, Na_2_O_3_Si·5H_2_O, combined trace elements, vitamin mix, buffer TES) and F/2 medium (NaNO_3_, NaH_2_PO_4_·2H_2_O, combined trace elements, vitamin mix, sea salt (Dupla Marin Natural Balance, Dohse Aquaristik GmbH & Co. KG, Grafschaft, Germany) were used to cultivate freshwater strains and the marine strain, respectively [43,44]. The strains were grown in three cabinets with different light conditions. The cabinet with standard features for growing various microalgae was considered the control cabinet, with white light (420–660 nm, peak wavelengths of 446, 517, and 630 nm, and intensity of 41 µmol_photons_ m^−2^ s^−1^). The other two cabinets were characterized by varied light conditions of blue light (420–540 nm, peak wavelength of 446 nm, intensity of 12 µmol_photons_ m^−2^s^−1^) and green light (470–570 nm, peak wavelength of 513 nm, intensity of 6 µmol_photons_ m^−2^s^−1^). Red light was not included, as the cryptophytes in this experiment did not grow under red light in preliminary tests. The light/dark cycle was 16 h: 8 h L/d, the temperature was set to 20 °C, and the bottles were mixed with gentle bubbling with 2% CO_2_ V/V air. Each strain had four replicates in each condition. The bottles were randomly distributed in the growth cabinets.

### 2.2. Biomass Production

Biomass productivity (BP: mg L^−1^day^−1^) was measured based on the oven-drying method. Briefly, Whatman GF/C filter papers (47 mm in diameter, ca 1.2 µm pore size) were dried at 105 °C overnight and then weighed. After filtering algal biomass using a diaphragm vacuum pump (KNF LABOPORT N 938.50, Freiburg, Germany), filter papers containing the algal biomass were dried and weighed again. Biomass dry weight was measured by subtracting the weight of empty filter paper from the filter paper including dry weight biomass. Finally, biomass productivity was calculated based on Equation (1) [45]:

(1) $$BP=\frac{\left(X_{1}-X_{0}\right)}{\left(t_{1}-t_{0}\right)}$$

where *X*_1_ and *X*_0_ are the concentration of biomass at the end (*t*_1_) and in the beginning (*t*_0_) of the experiment.

### 2.3. Phycoerythrin Content

Cryptophyte samples (40 mL each) were centrifuged for 10 min at 2000× *g* (Heraeus Multifuge 1 S-R, Kendro Laboratory Products, Hamburg, Germany). The pellets were suspended in 10 mL of 0.1 M phosphate buffer and stored at −20 °C to rupture the cells and release the phycoerythrin. Next, after one freeze-thaw cycle at 5 °C for 24 h, the thawed samples were centrifuged at 4000× *g* for 15 min to remove cell debris, and the supernatants were collected in clean tubes for pigment analysis [46]. 

The absorbance (A) of purified extracts was measured from 280 to 750 nm using spectrophotometry (Shimadzu UV-2401PC, Kyoto, Japan) and a 1-cm cuvette (d) against phosphate buffer as a blank. The phycoerythrin concentration (c) (in µg/L) was calculated using Equation (2) [46]:

(2) $$c=\frac{A}{ɛd}\times MW\times \frac{V_{buffer}}{V_{sample}}\times 10^{6}$$

where *A* is derived from subtracting the absorbance at 750 nm from the absorbance maximum of the phycoerythrin peak, ε is the molar extinction coefficients (2.41 × 10^6^ L mol^−1^cm^−1^), MW is the molecular weight of phycoerythrin (240,000 g mol^−1^), and *V_buffer_* and *V_sample_* are the volume of the buffer and sample, respectively. In addition, the purity index (PI) of phycoerythrin was measured by using Equation (3) [47]:

(3) $$PI=\frac{A_{max\;PE}}{A_{280}}$$

where *A_max PE_* is the maximum absorbance of phycoerythrin and *A*_280_ is the absorbance of phycoerythrin at 280 nm.

### 2.4. Phenol Content

Dry biomass was mixed with the solvent (methanol), and the mixture was placed in an ultrasonic bath (Branson 8510, Brookfield, CT, USA) at 50 Hz and 37 °C for 15 min. After incubating samples for an hour at room temperature, the mixture was centrifuged at 4000× *g* for 15 min, according to [48,49].

Th measurement of total PC was carried out by the Folin–Ciocalteu (FC) method. Folin–Ciocalteu’s phenol reagent (1.5 mL) and Na_2_CO_3_ (7.5% *w*/*v*: 1.2 mL) were added to the sample extract (300 µL). After incubating in the dark for 30 min at room temperature, the absorbance was read at 765 nm. Gallic acid (GA) was used as a reference for the standard curve, and PC was expressed as GAE in mg per g of algae dry weight (mg GAE/g DW), according to [11,49].

### 2.5. Exopolysaccharide Content

A combination of physical and mechanical methods was used to extract EPS. Mixtures of freeze-dried biomass and 5 mL of deionized water were shaken for 20 min. Samples were then centrifuged at 4000× *g* for 15 min, and pellets were collected for further analysis. Wet biomass was suspended in 5 mL of a 0.05% NaCl solution and incubated in an overhead shaker (New Brunswick Scientific C25KC, Enfield, CT, USA) for one hour at 60 °C. After the sonication of samples in an ultrasonic bath for 10 min at 100 W and 20 °C, the treated suspensions were centrifuged for 15 min at 4000× *g*. Finally, supernatants were lyophilized (Christ, Beta 2-8 LSCbasic, Ottobeuren, Germany) for 48 h at −60 °C and 0.6 mbar, and the weight of total EPS was determined gravimetrically, according to [50,51].

### 2.6. Antioxidant Activity

The antioxidant activity of the extracted bioactive compounds was determined using a DPPH (2,2-diphenyl-1-picrylhydrazyl) solution (0.1 mM). Briefly, 1 mL of the extract was mixed with 3 mL of a DPPH solution. The mixture was incubated in the dark for 30 min, after which the absorbance (A) was read at 517 nm. The percentage inhibition was calculated using Equation (4):

(4) $$\%\;inhibition=\frac{\left(A_{DPPH}-A_{sample}\right)}{A_{DPPH}}\times 100$$

The IC_50_ value (concentration of sample required to scavenge 50% of free radicals) was estimated using a line curve for % inhibition and different concentrations of compounds [48]. 

### 2.7. Statistics

The statistical analyses were performed with the IBM SPSS 26 Statistical package (SPSS Inc., Chicago, IL, USA). The data were analyzed using a two-way MANOVA to determine the effect of light and species changes and their interaction on the studied compounds, a one-way ANOVA to assess the effect of change in light on the studied compounds in each strain separately, and a Tukey post-hoc test. The significance level was set to *p* < 0.05.

## 3. Results

### 3.1. Biomass Production

**Figure 1:**
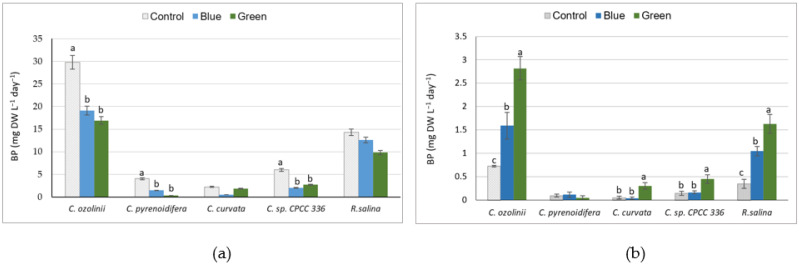
The studied cryptophyte strains were grown under various light conditions (control (white), blue, and green): (**a**) Biomass production (BP; mg dry weight L^−1^day^−1^); (**b**) biomass production per light unit (µmol_photons_ m^−2^s^−1^). Significant differences between samples are indicated with different letters as determined by ANOVA comparison (*p* < 0.05).

Among the studied strains, *C. ozolinii* produced significantly higher biomass than the other strains (*p* < 0.05), followed by *R. salina*; the dry biomass of *C. ozolinii* was almost 1.5, 6, 8.5, and 14.5 times higher than the biomass of *R. salina*, *C*. sp. CPCC 336, *C. pyrenoidifera*, and *C. curvata*, respectively. The dry biomass under the control light with high intensity was significantly higher than under blue and green lights with lower intensities (*p* < 0.05, Figure 1a), excluding *C. curvata* and *R. salina*, for which there was no significant difference in dry biomass under different light conditions. However, when the light intensity was taken into account, the biomass productivity of the studied cryptophytes was significantly higher under green light, excluding *C. pyrenoidifera* (Figure 1b). The growth rates were higher under control conditions compared to blue or green lights for all strains except *C. curvata*. For *C. curvata*, there was no significant difference in growth rate under different light conditions (Supplementary Figure S1).

### 3.2. Biochemical Compositions

#### 3.2.1. Phycoerythrin Extraction Yield

**Figure 2:**
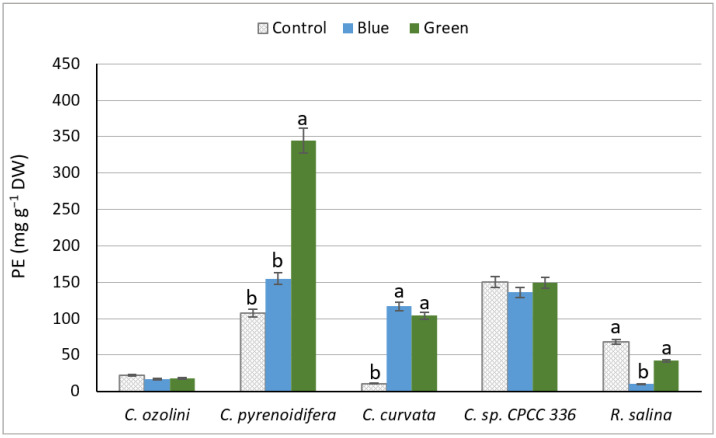
Phycoerythrin (PE) concentration (mg PE g^−1^ dry weight) extracted from five different cryptophyte species cultivated under various light lights (control (white), blue, and green lights with different intensities). Significant differences between samples are indicated with different letters as determined by ANOVA comparison (*p* < 0.05).

There was a significant difference between strains in PE production. The highest PE content was produced by *C. pyrenoidifera* under green light (*p* < 0.05), whereas the lowest PE content was detected for *R. salina* under blue light (Figure 2). The light conditions did not affect the PE content of *C. ozolinii* or *C.* sp. CPCC 336 (*p* > 0.05). However, *C. pyrenoidifera* had significantly higher PE content under green light (*p* < 0.05), and the PE content of *C. curvata* was higher under the low-intensity lights (blue and green) than under the high-intensity control light (*p* < 0.05). For the marine *R. salina*, there was no significant difference in PE content between control and green lights (*p* < 0.05), but the PE content was significantly higher under control and green lights than under blue light (*p* > 0.05). In general, strains grown under green light had significantly higher PE content than control and blue lights (Figure 2). 

The purity index (PI) is an indicator of the degree of purification to reveal the PE quality and function for different applications. *C. ozolinii* had the highest PI under green light, but in *C. curvata*, the highest PIs were observed under blue and green light (*p* < 0.05). The PI in *C. pyrenoidifera*, *C*. sp. CPCC 336, and *R. salina* did not respond to different light conditions (*p* > 0.05; Table 1). In general, the PI of phycoerythrin was higher for cultures grown under green light than the PI of those exposed to the control light (Table 1).

#### 3.2.2. Phenol Content

**Figure 3:**
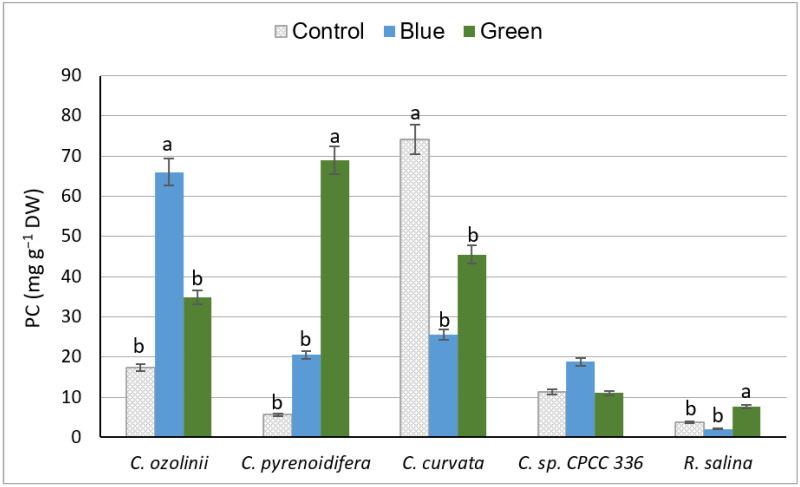
Total phenol content (PC: mg GAE g^−1^ DW) of the five studied cryptophyte species cultivated under different light conditions (control (white), blue, and green lights with different intensities). Significant differences between samples are indicated with different letters as determined by ANOVA comparison (*p* < 0.05).

The total phenol content varied under different light conditions depending on the studied cryptophyte species. Of the studied strains, *C. curvata*, *C. pyrenoidifera*, and *C. ozolinii* produced the highest amount of PC under control white, green, and blue lights, respectively (*p* > 0.05), whereas the lowest PC was detected from *R. salina* (*p* < 0.05; Figure 3). Although there was no significant difference in PC of *C.* sp. CPCC 336 grown under planned light conditions, *C. pyrenoidifera* and *R. salina* had significantly higher PC under green light (*p* < 0.05), whereas *C. curvata* had the highest PC under the control light. In *C. ozolinii*, PC was significantly higher under blue light than under control or green lights (*p* < 0.05; Figure 3).

#### 3.2.3. Exopolysaccharide Content

**Figure 4:**
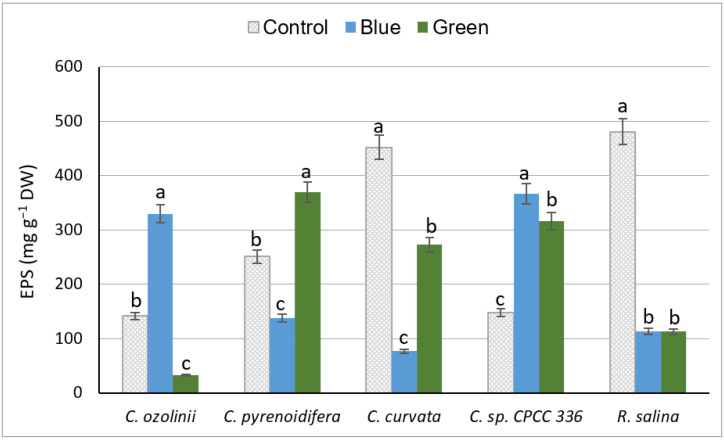
EPS content (mg g^−1^ DW) of studied cryptophyte species cultivated under different light intensities and wavelengths (control (white), blue, and green lights with different intensities). Significant differences between samples are indicated with different letters as determined by ANOVA comparison (*p* < 0.05).

Similar to PE and PC, a species-specific pattern was noticeable in EPS under different light conditions. Among the studied cryptophyte strains, *C. curvata* grown under control white light had a significantly higher amount of EPS (*p* < 0.05), whereas the lowest EPS content was seen in *C. ozolinii* under green light (*p* < 0.05). Of the strains, *C. ozolinii* and *C*. sp. CPCC 336 had the highest EPS under blue light, but *C. pyrenoidifera* had the highest under green light (*p* < 0.05; Figure 4). On the contrary, for *C. curvata* and *R. salina*, cultures exposed to control light had the highest EPS contents (*p* < 0.05; Figure 4).

### 3.3. Antioxidant Activity of Derived Bioactive Compounds

Antioxidant activity was described as IC_50_ (µg mL^−1^; half maximal inhibitory concentration), which indicates the concentration of antioxidant required for 50% inhibition of DPPH free radicals. Based on this approach, a lower IC_50_ value has a higher antioxidant activity. The antioxidant activity was influenced by the species and light conditions. *C. curvata* had the highest antioxidant activity among the studied strains, and in general, blue and green lights provided the optimal conditions to produce biomolecules with high antioxidant activity. 

PE showed a significant difference in antioxidant activity between species and light conditions. Antioxidant activities of PE under blue and green lights were significantly higher than under control light (*p* < 0.05) in all strains except *C. curvata* (Table 2). There were no significant differences between the antioxidant activity of PE under blue and green lights in *R. salina*, *C. pyrenoidifera*, and *C*. sp. CPCC 336 (*p* > 0.05; Table 2).

There was a species-specific pattern to the antioxidant activity of PC derived from different species under diverse light conditions. The highest PC antioxidant activity was detected in *C. curvata* grown under both control and green lights, whereas the lowest antioxidant activity was in *C. ozolinii* cultivated under control light. Similar to *C. ozolinii*, the *C*. sp. CPCC 336 had lower antioxidant activity under control light than blue or green light. The *C. pyrenoidifera* had higher antioxidant activity under control and blue lights than under green light, whereas antioxidant activity in *R. salina* was higher under blue light than under control and green lights (Table 2). 

Based on the results related to the antioxidant activity of EPS, there was a significant difference between the studied species regarding the antioxidant activity of derived EPS (*p* < 0.05). The EPS from *C. ozolinii* and *C. curvata* displayed the highest antioxidant activity, while *R. salina* had the lowest (Table 2).

## 4. Discussion

### 4.1. Effect of Light Quality on Biomass Production

Our results show that the influence of light on biomass productivity varies between species. The highest dry biomass in this study was harvested from *C. ozolinii*, followed by *R. salina*. One of the species features affecting the light requirements, photosynthetic characteristics, and carbon fixation patterns is the algal cell size [52]. Species with smaller cell sizes have shorter cell cycles, and consequently, higher growth rates and biomass productivity [53]. The higher growth rate and biomass of *R. salina* with smaller body size (5 µm) compared to the larger species *C. curvata* and *C. pyrenoidifera* (~32–61 µm) [54] support this hypothesis. 

Light quality has an effect on algal biomass gain, and the most suitable wavelength (color) may differ in accordance with algal genus [55]. Among the species of cryptophytes cultivated under different wavelengths, *Guillardia theta*, *R. salina*, *Proteomonas sulcata*, *Storeatula* sp., *Hemiselmis andersenii*, *H. cryprochromatica*, and *Cryptomonas* CCAP 979/67 have been shown to have their maximum growth rates under blue light [56,57]. These findings are contrary to our study, where the two experimental conditions—blue and green lights—showed lower biomass production compared to the control (white) light. However, similar to our study, the highest biomass production of *Rhodomonas* sp. Was reported under white light by Latsos et al. [24]. In relation to the effect of light intensity on biomass yield, all studied cryptophyte species grew rapidly under control light with higher intensity than under green and blue lights. However, when considering the light intensity, the biomass gain per light unit was generally highest under green light. Several studies have reported the direct effect of light intensity on microalgal growth. For example, *R. salina* grown under white and blue lights displayed higher growth and biomass productivity under higher light intensities (60 and 80 µmol m^−2^ s^−1^compared to 8 µmol m^−2^ s^−1^) [24]. However, the effects of light intensity on growth were not in the scope of our study.

### 4.2. Effect of Light Quality on Bioactive Compounds Content

#### 4.2.1. Phycoerythrin

Our results show that the influence of light on phycoerythrin (PE) productivity varies between species. This could be due to autoregulation processes carried out by photosynthetic mechanisms to balance the accumulation of light and store the needed energy for microalgal growth [24,58]. When comparing the biomass production and PE concentrations of *C. ozolinii* and *R. salina*, we noted that the highest dry biomass contained the lowest amount of PE. On the other hand, *C. pyrenoidifera*, *C. curvata*, and *C*. sp. CPCC 336 showed the lowest biomass production but produced a considerable amount of PE. In fact, the results suggest that for the cultures of *C. ozolinii* and *R. salina*, energy had been exploited for growth, but in cultures of *C. pyrenoidifera*, *C. curvata*, and *C*. sp. CPCC 336, energy was used for pigment synthesis. In a previous study, the PE concentration of a *Chroomonas* strain was observed to be higher under blue-green light in comparison to white-light treatment [59,60]. This finding is similar to the results of *C. curvata* in our study. Moreover, *Hemiselmis andersenii*, *Hemiselmis cryprochromatica*, and *Chroomonas mesostigmatica* grown under green light also produced higher PE compared to white-, blue-, and red-light treatments [56], similar to *C. pyrenoidifera* in our study. From the marine cryptophytes, *R. salina* grown under green light has been reported to produce more PE compared to blue and white lights [24,56]. However, in our study, the PE production of *R. salina* did not differ between the control white light and green light, although PE production under both green and white lights was higher than in blue light. Overall, low light conditions increase the efficiency of algal cells to gain the photons needed for photosynthesis to produce more pigments [61,62], also shown in *C. pyrenoidifera* and *C. curvata* in our study. 

**PE purification**: The purity index (PI) or purification grade of phycoerythrin shows PE quality and the application potential for different industries. PI is considered to be food grade when it is above 0.7, reactive grade when it is above 3.9, and analytical grade when it is higher than 4 [63]. The extraction method has a considerable effect on PE purity. In this study, we used the freeze–thaw method and potassium phosphate buffer, which result in the highest PE content and high purity [64]. In the present study, the PI ranged from approximately 2 to 13, and was highest under green light. The PIs have been studied for a wide range of red algae, showing values between 3 and 7 [65,66,67,68,69]. Similar to the red algae, the PI index of *C. pyrenoidifera*, *C*. sp. CPCC 336, and *C. ozolinii* (under green light) in our study is noticeable (PI above 4), suggesting they could be promising species for producing high-quality and efficient PE for medical applications. PE with a high PI index can be used for cancer treatment in photodynamic therapy as a model photosensitizer, as well as in pharmaceuticals (the drug grade of PI is 3) [66]. *C. curvata* and *R. salina* with grades under 3.9 could be useful as a colorant in the food and cosmeceutical sectors.

#### 4.2.2. Phenolic Content

Based on our results, there is an inverse relationship between algal biomass and phenol accumulation. While almost all studied cryptophytes grew effectively under control light, they produced more phenolic compounds under blue or green lights. Light can influence phenol accumulation by its effect on algal growth and metabolism. Strains with low biomass production such as *C. pyrenoidifera* and *C. curvata* contained high PC, whereas strains such as *R. salina* with high biomass production had low PC. This may be because dilute cultures have minimum self-shading, and higher light penetration leads to an increase in PC production. In addition, this could be a result of pH affecting algal growth—pH levels affect algal growth by changing the carbon availability in photosynthesis or disturbing the cell membrane process in algal cultures. This contributes to organic accumulation by algae [70]. There is also a connection between the growth phase and bioaccumulation of biochemical compounds: Over the exponential phase, algal species allocate energy towards growth, thus the accumulation of biochemical compounds begins during the stationary phase [71]. As our samples were collected during the exponential phase, the growth pattern and rate in this phase may have an effect on the accumulation of biochemical compounds. 

Since studies on the effect of light quality on algal PC are rare, we compare our results with certain studies on PC of plants. PC extracted from *Gynura procumbens* was higher under blue light than white light [72], similar to our results of *C. ozolinii*, *C. pyrenoidifera*, and *C*. sp. (CPCC 336). *Pachyrhizus erosus* cultured under different LED lights produced more PC under blue light, followed by white and green lights [73], as did *C. ozolinii* and *C*. sp. (CPCC 336) in our study. This is likely because blue light affects the activity of the phenylalanine ammonialyase enzyme (PAL), a key enzyme in the phenol synthesis pathway and accumulation [74,75]. 

#### 4.2.3. Exopolysaccharides

The results of our study indicated a species-dependent response to the effect of light changes on EPS production, similar to marine diatom studies showing that the process of EPS execration is species-specific [76]. Although our strains grew better under control light, some of them (*C. ozolinii*, *C. pyrenoidifera* and *C*. sp. CPCC 336) produced the highest quantity of EPS under blue and green lights. Similar to our results from *C. ozolinii* and *C*. sp. CPCC 336, blue light favored the EPS production of the red algae *Porphyridium cruentum* and cyanobacterium *Nostoc flagelliforme* [77,78]. On the other hand, blue light was the least favorable for EPS production of *P. sordidum* [66] and our *C. pyrenoidifera* and *C. curvata*. Similar to *C. curvata* and *R. salina* in our study, *P. purpureum*, *P. sordidum*, and *N. calcicola* had their highest EPS content under white light [79,80]. Green light led to the highest EPS production in our *C. pyrenoidifera*, and similar results have been obtained for *P. cruentum* [81]. 

There is a hypothesis that favorable circumstances for algal growth differ from those for EPS production [82]. The effect of light wavelengths on the synthesis of EPS might be induced through carbon metabolism, which depends on the availability of energy under various light conditions [78,83]. EPS accumulation is governed by the ratio of carbon fixation and utilization [84], and photosynthetic activity is also dependent on nutrient concentrations; thus, the cellular nutrient status can then affect EPS production [82]. The positive impact of a lower nitrogen concentration at the end of the growth phase on EPS production might lead to an increase in the C:N ratio, and therefore, enhanced carbon interpolation into polymers [85]. Additionally, non-optimal growth conditions could affect EPS excretion, and thus the reduction of growth of these species under blue and green lights could be a result of oxidative stress leading to increasing EPS as a defense mechanism [82,86]. Studies on *Nostoc flagelliforme* demonstrated that light quality has a direct effect on EPS biosynthesis through changes in intracellular reactive oxygen species (ROS) levels [87]. 

### 4.3. The Effect of Light Quality on Antioxidant Activity

Although the influence of light quality on levels of antioxidant activity is clear in our study, five studied cryptophytes produced biochemical compounds with remarkable antioxidant activity (<50 µg mL^−1^) according to the classification of antioxidant activity power outlined by Jun et al. [88]. *C. curvata* contained the most active antioxidants, particularly in phenolic compounds. The antioxidant activity of the studied compounds varied significantly among light treatments. Green light induced strong antioxidant activity, specifically for PE and EPS. The free radical scavenging potential of PC was different from PE and EPS, such that the species had mixed reactions to light-quality changes in terms of antioxidant properties. The highest antioxidant activity of PC was under blue light for *C. ozolinii* and *R. salina*, under green light for *C. curvata* and *C*. sp. CPCC 336, and under the control light for *C. pyrenoidifera*.

There is no literature on the effect of light quality on antioxidant activity of algal biochemical compositions. To date, studies have only focused on the effect of different light wavelengths on antioxidant enzyme activity for coral-associated dinoflagellates, demonstrating the high activity of superoxide dismutase (SOD) and catalase (CAT) under blue light [89], in agreement with some results of this study. 

### 4.4. Commercial Perspective

Our aim was to study the effect of light color on the production of biochemical compounds of cryptophyte algae to evaluate their potential as natural sources of sustainable components, e.g., for the food industry. To fulfil this aim, quantities of isolated components from studied cryptophytes have been compared to well-known microalgal species used commercially. 

The phycoerythrin (PE) content in the studied cryptophytes ranged from approximately 2 to 34.5% of DW. The best PE producer—*C. pyrenoidifera* (35% of DW, under green light)—is superior when compared to the red algal genus *Porphyridium*, a well-known genus for PE production (42% of DW) [90]. Additionally, the purity of the *C. pyrenoidifera* PE was high, indicating that this species could be considered a possible alternative commercial source of PE. *C. curvata*, *C. pyrenoidifera*, and *C. ozolinii* could be used as a rich source of polyphenols, as their PC contents (~7.5, 7, and 6.5% PC of DW, respectively) are comparable to the already commercialized algae *Haematococcus pluvialis*, *Gracilaria gracilis*, *Spirulina maxima*, *Chlorella minutissima*, and *Porphyridium cruentum* (PC ~ 7.5, 6.5, 1.3, 1.8, and 3.4% of DW, respectively [91,92,93,94]). EPS are one of the algal polysaccharides with various biological activities. The studied cryptophytes have demonstrated that they could also be a promising source of EPS, with approximately 14 to 48% of EPS in DW, in comparison to *P. cruentum*, *Chlamydomonas mexicana*, and *Chlorella* sp. with approximately 45, 25, and 17% of DW, respectively [95,96]. Regarding their antioxidant capacity, the studied biochemical compounds were highly active (IC50 < 50 µg mL^−1^). They could therefore be used as an alternative natural source of antioxidants to retard the oxidation process by scavenging free radicals. 

## 5. Conclusions

Based on our observations, changes in light quality significantly affect cryptophyte biomass productivity and chemical compositions, including PE, PC, and EPS. Importantly, species-specific responses to changes in light quality should not be ignored. Although the maximum biomass productivities were attained under the control white light, the optimal light conditions for the production of the studied bioactive compounds with high quantity and antioxidant activity were obtained under blue or green light. Therefore, a possibly useful practice for the production of bioactive compounds might be to first grow the biomass under white light and then switch to blue or green light. Finally, when comparing the number of derived biomolecules from the studied cryptophytes with commercially used species, it should be noted that cryptophytes could be considered promising candidates to produce natural biochemical products.

## Figures and Tables

**Table 1:** Purity index (PI) of phycoerythrin extracted from studied cryptophyte algae cultured under three different LED lights.

	*C. ozolinii*	*C. pyrenoidifera*	*C. curvata*	*C*. sp. CPCC 336	*R. salina*
LED Lights	PI	PI	PI	PI	PI
control	2.7 ^b^	5.4	0.2 ^b^	4	3.1
Blue	2.2 ^b^	8.6	3.9 ^a^	5.7	2.4
Green	9.5 ^a^	13	3 ^a^	6.4	3.5

Values in the same columns indicate statistically significant difference (ANOVA *p* < 0.05) of each strain under various lights, represented with letters ^a,b^.

**Table 2:** Antioxidant activity (IC50, µg mL^−1^) of isolated chemical components from studied cryptophyte algae cultured under three different LED lights.

	*C. ozolinii*			*C. pyrenoidifera*			*C. curvata*			*C*. sp. CPCC 336			*R. salina*		
	IC50			IC50			IC50			IC50			IC50		
LED Lights	PE	PC	EPS	PE	PC	EPS	PE	PC	EPS	PE	PC	EPS	PE	PC	EPS
Control	35.5 ^c^	148 ^c^	9.7 ^c^	70 ^b^	13 ^a^	12.2 ^a^	10	1.1 ^a^	10 ^b^	97 ^b^	30.5 ^c^	18.6 ^b^	140 ^b^	8.9 ^b^	35 ^c^
Blue	25 ^b^	7.3 ^a^	6 ^b^	30 ^a^	15.7 ^a^	17.7 ^b^	14	3.3 ^b^	6.3 ^a^	41 ^a^	12 ^b^	18.7 ^c^	18 ^a^	4.3 ^a^	27 ^b^
Green	14.5 ^a^	33 ^b^	3.5 ^a^	26 ^a^	19 ^b^	11 ^a^	13.5	0.93 ^a^	6.3 ^a^	40 ^a^	7 ^a^	15 ^a^	17 ^a^	10 ^b^	20 ^a^

Values in the same columns indicate statistically significant difference (ANOVA *p* < 0.05), represented with letters ^a–c^.

## Data Availability

The raw data that support the findings of this study are available on request from the corresponding author [M.A.].

## Supplementary Materials

The following supporting information can be downloaded at: https://www.mdpi.com/article/10.3390/biology11081112/s1, Figure S1: The growth rates (µ) of five studied cryptophyte strains cultivated under different light LEDs (white, blue and green).
