# Supplementary material for: Promising Biomolecules with High Antioxidant Capacity Derived from Cryptophyte Algae Grown under Different Light Conditions
Source: Biology (Basel). 2022 Jul 26;11(8):1112. doi: 10.3390/biology11081112 (PMC9331842; doi:10.3390/biology11081112)
Supplement: Supplementary file 1 [file biology-11-01112-s001.zip › biology-1815224-supplementary.pdf]

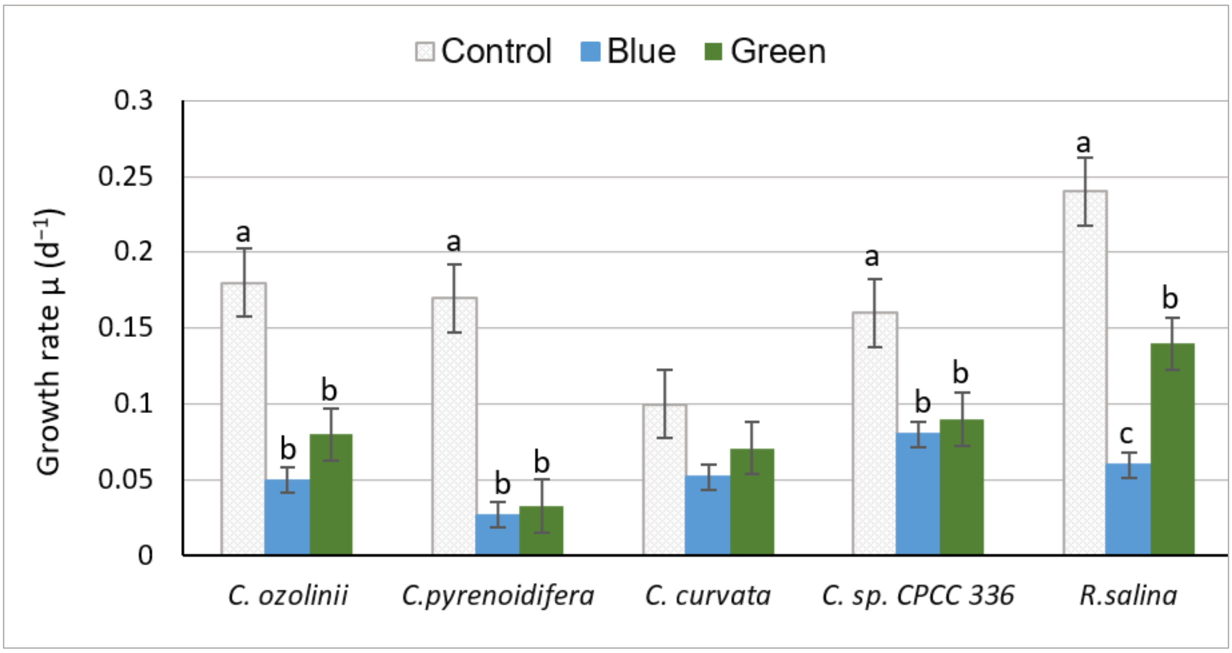

Figure S1: The growth rates (μ: divisions day^{-1}) of five studied cryptophyte strains cultivated under different light LEDs (white, blue and green). Significant differences between samples are indicated with different letters as determined by ANOVA comparison ($p < 0.05$).
